# A Meta-Analysis of the Analgesic Efficacy of Single-Doses of Ibuprofen Compared to Traditional Non-Opioid Analgesics Following Third Molar Surgery

**DOI:** 10.3390/ph14040360

**Published:** 2021-04-14

**Authors:** Lorenzo Franco-de la Torre, Norma Patricia Figueroa-Fernández, Diana Laura Franco-González, Ángel Josabad Alonso-Castro, Federico Rivera-Luna, Mario Alberto Isiordia-Espinoza

**Affiliations:** 1 Instituto de Investigación en Ciencias Médicas, Departamento de Clínicas, División de Ciencias Biomédicas, Centro Universitario de los Altos, Universidad de Guadalajara, Tepatitlán de Morelos 47620, Mexico; lorfran8888@hotmail.com (L.F.-d.l.T.); diana.franco5288@alumnos.udg.mx (D.L.F.-G.); 2 Departamento de Cirugía Oral y Maxilofacial, Facultad de Odontología, Universidad Autónoma de Baja California, Campus Mexicali 21040, Mexico; nfigueroa@uabc.edu.mx (N.P.F.-F.); rivera_l03@hotmail.com (F.R.-L.); 3 Departamento de Farmacia, División de Ciencias Naturales y Exactas, Universidad de Guanajuato, Guanajuato 36250, Mexico; angeljosabad@hotmail.com

**Keywords:** ibuprofen, non-steroidal anti-inflammatory drugs, dental pain, adverse effects, third molar surgery

## Abstract

The purpose of this systematic review was to determine the analgesic efficacy and adverse effects of ibuprofen in comparison with other traditional non-opioid analgesics after third molar surgery. A total of 17 full texts were identified in PubMed and assessed using the Cochrane Collaboration’s risk of bias tool by two independent researchers. The sum of pain intensity differences, total pain relief, the overall evaluation, the number of patients requiring rescue analgesics, and adverse effects were collected. Data were analyzed using the Review Manager Software 5.3. for Windows. A total of 15 articles met the criteria. The qualitative and quantitative analysis showed that ibuprofen is more effective to relieve post-operative dental pain than acetaminophen, meclofenamate, aceclofenac, bromfenac, and aspirin. Moreover, ibuprofen and traditional non-steroidal anti-inflammatory drugs have a similar safety profile. In conclusion, ibuprofen 400 mg appears to have good analgesic efficacy and a safety profile similar to other traditional non-steroidal anti-inflammatory drugs after third molar surgery.

## 1. Introduction

The most common signs and symptoms after lower third molar extraction are post-surgical pain, facial swelling, and trismus [1,2,3,4]. These complications are closely related to soft tissue trauma and osteotomy during the surgical procedure [4,5]. The most intense postoperative pain occurs during the second and sixth hours after the extraction of the third molar, and some episodes can even occur during the next seven days [6,7]. Swelling and trismus reach their critical point between the second and third day and disappear approximately 1 week after surgery [8,9]. 

Non-steroidal anti-inflammatory drugs (NSAIDs) are the first option to control complications in oral surgery [10]. Particularly, ibuprofen is one of the most used drugs by general dentists and specialists worldwide for the control of inflammatory complications after third molar removal [11,12]. Besides, other NSAIDs are also broadly used for this purpose (e.g., diclofenac and paracetamol) [13,14,15]. This kind of drug induces its therapeutic and adverse effects by the inhibition of the cyclooxygenase enzyme [10,11,12,13,14,15].

Ibuprofen is widely used for pain management following third molar surgery [11,12]; however, there is not a practical guide to help the clinician decide whether to use this drug or another one that is available [11,12]. We found only one meta-analysis comparing ibuprofen with another drug. That study demonstrated that ibuprofen is more effective to relieve pain compared to acetaminophen in third molar removal [15]. Nonetheless, there is not a qualitative review of the analgesic efficacy of ibuprofen in comparison with other analgesics (different from acetaminophen) nor a quantitative assessment integrating the data to make a decision about which analgesic to use after third molar surgery. For that reason, the purpose of this systematic review was to determine the analgesic efficacy and adverse effects of ibuprofen in comparison with other traditional non-opioid analgesics following wisdom teeth removal.

## 2. Material and Methods

### 2.1. Study Registration

This systematic review (and meta-analysis) was registered on 18 December 2020, in the National Institute of Health Research from the University of York, United Kingdom (PROSPERO ID: CRD42021227135).

### 2.2. Selection Criteria

The inclusion criteria were as follows: Clinical trials comparing single-dose of ibuprofen versus non-opioid analgesics in lower wisdom teeth extraction using parallel or crossover design, including patients of both sexes and over 15 years old, reporting the effect of ibuprofen alone, and articles in English or Spanish. A loss to follow-up of more than 20% of those entered was the only exclusion criteria.

### 2.3. Article Digital Searching

The PubMed database browser was used to detect scientific articles that compared single-doses of ibuprofen with non-opioid analgesics. Two information filters from this browser were used: Type of article—selecting the boxes: clinical trial—and randomized controlled trial—and language—employing the boxes: English and Spanish. All articles found without date restriction until 31 December 2020, were included. The keywords used were the following: “Ibuprofen”; “Non-steroidal anti-inflammatory drugs”; “Diclofenac”; “Ketorolac”; Meloxicam”; “Piroxicam”; “Acetaminophen”; “Ketoprofen”; “Metamizole”; “Indomethacin”; “Naproxen”; “Third molar surgery”; “Oral surgery”; “Maxillofacial surgery”. 

### 2.4. Quality Assessment

The evaluation of bias was performed with the Cochrane Collaboration’s risk of bias tool [16,17,18]. Studies rated low-risk (green color circle) or medium-risk (yellow color circle) of bias according to the summary figure were designated of high quality. Two researchers did the full-text evaluations and the differences were resolved with the participation of a third researcher [19,20].

### 2.5. Data Collection

The primary results were as follows: Sum of pain intensity differences (SPID) at 2 and 6 h after surgery, total pain relief (TOTPAR or TOPAR) at 2, 4, 6, and 8 h following surgery, and overall evaluation (number of patients reporting a good, very good, and excellent effect). Moreover, the secondary outcomes were the number of patients requiring rescue analgesics within the first 24 post-surgical hours and adverse effects. When a clinical trial had two or more ibuprofen groups or when the active controls were several NSAIDs, the data were summed and evaluated in a single group (e.g., for the evaluation of adverse effects in the Forbes et al., 1991 study, there were two ibuprofen groups (200 and 400 mg) and two meclofenamate groups (50 and 100 mg)) [21]. On the other hand, the percentages and proportions were converted to absolute numbers [21].

### 2.6. Statistical Analysis

The Review Manager Software 5.3. for Windows was used to carry out the pooled data analysis. The SPID and TOTPAR were evaluated using the inverse variance statistical method and means difference. The overall evaluation, the number of patients requiring rescue analgesics, and the adverse effects were assessed with the Mantel–Haenszel test and Odd Ratio (OR). The I2 test was interpreted according to Higgins and Green, 2011 [18,22]. A *p*-value ≤ 0.05 was considered significant. 

## 3. Results

### 3.1. Digital Search and Assessment of Bias

**Figure 1:**
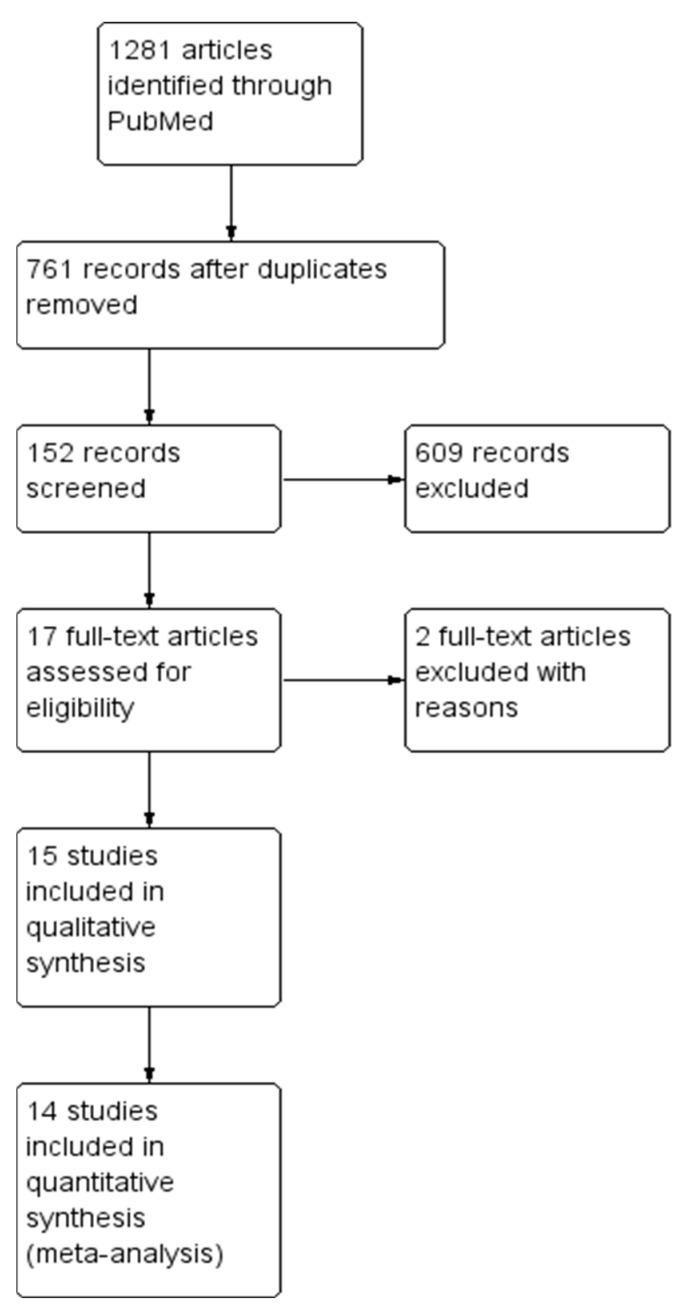
Study flow diagram.

A total of 1281 articles were identified in PubMed, of which 17 were fully evaluated with the Cochrane Collaboration’s risk of bias tool and, finally, only 15 met the requirements of our study [23,24,25,26,27,28,29,30,31,32,33,34,35,36,37] (Figure 1). 

### 3.2. Qualitative Evaluation

**Figure 2:**
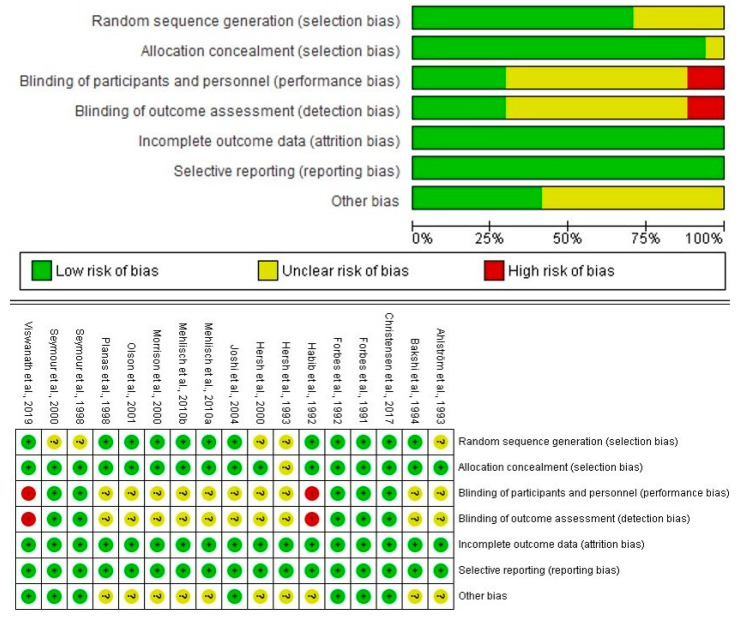
Evaluation of bias of the full-text articles.

The assessment was done with 15 articles. Six clinical studies were in favor of ibuprofen, two reports presented similar analgesic effects, three assays showed no conclusions about the analgesic efficacy of ibuprofen, and four clinical trials informed a result against this drug [23,24,25,26,27,28,29,30,31,32,33,34,35,36,37] (Figure 2; Table S1).

### 3.3. Analgesic Efficacy

The quantitative analysis of the SPID showed that ibuprofen was superior to acetaminophen at 2 and 6 h after surgery. Furthermore, similar scores of the SPID were observed for ibuprofen and ketoprofen 2 h following surgery. However, at 6 post-surgical hours, ibuprofen had lower pain scores than ketoprofen (Table S2).

**Figure 3:**
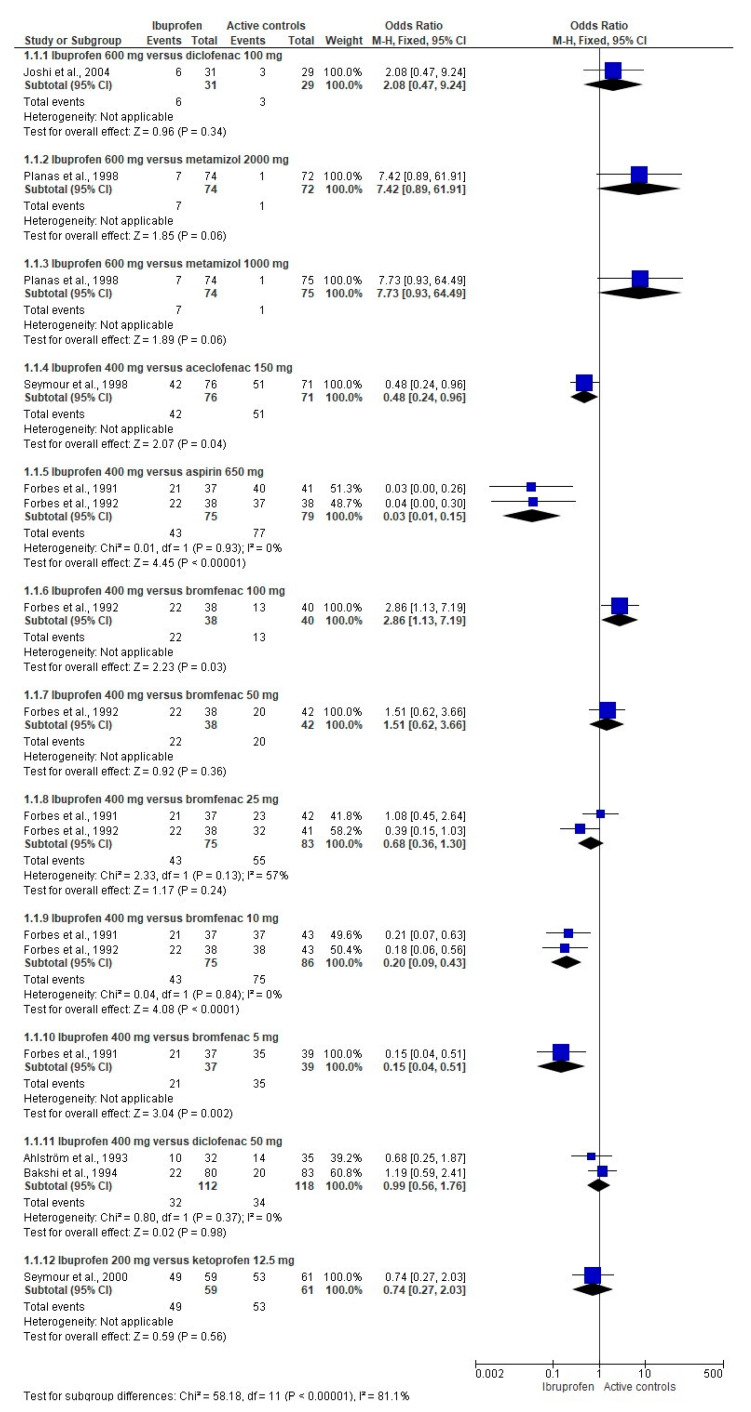
Meta-analysis of the number of patients using the rescue medication after third molar extraction.

**Figure 4:**
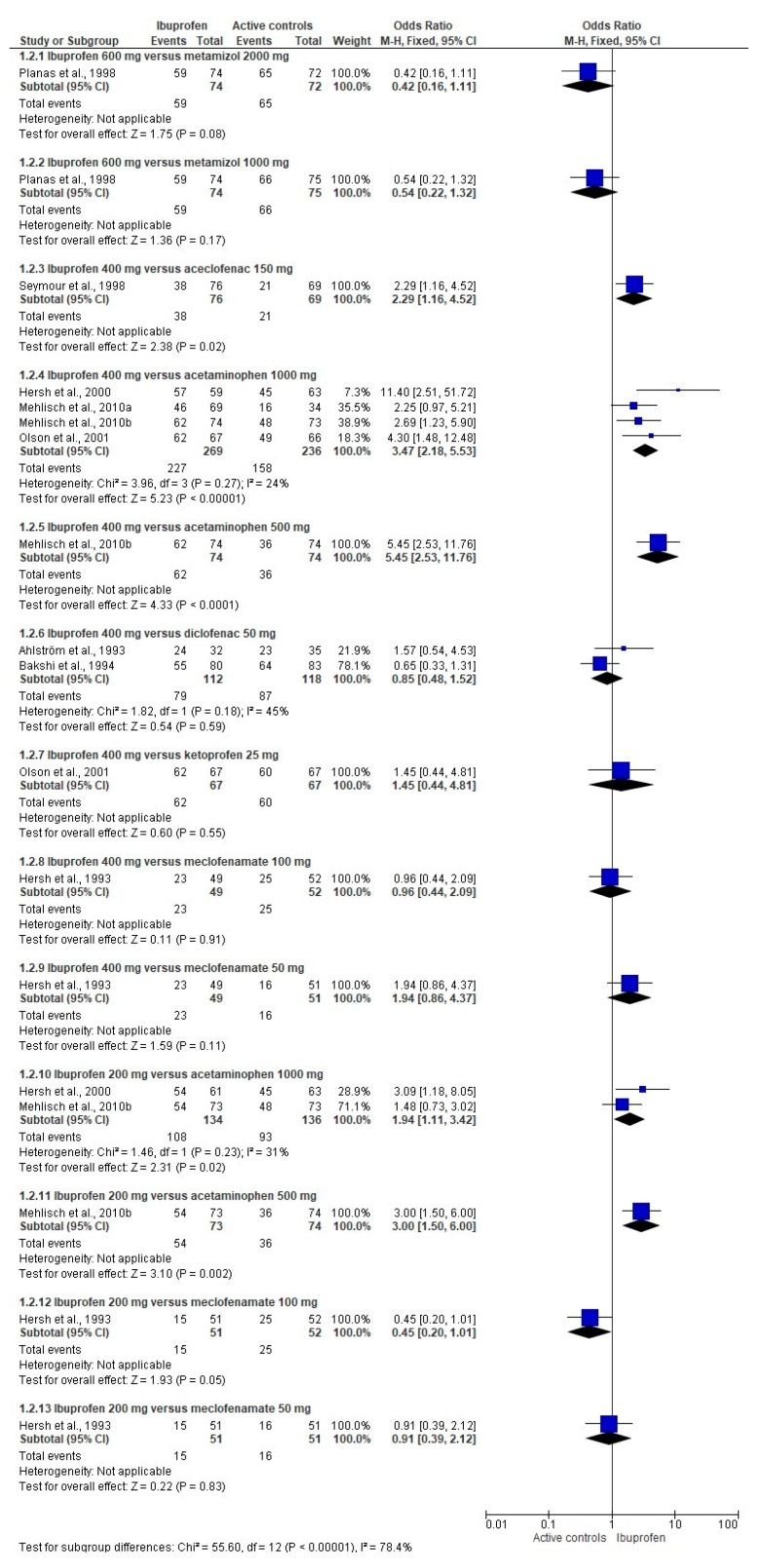
Combined analysis of the overall evaluation of the study medication following wisdom teeth surgery.

The TOTPAR meta-analysis confirmed these findings (Table S3). In addition, a lower dose of ibuprofen was inferior to a low dose of ketoprofen at 4 and 6 post-operative hours. The TOTPAR scores were better for the ibuprofen when compared to meclofenamate at 8 post-surgical hours (Table S3). The pooled analysis showed that ibuprofen decreases the number of patients using the rescue medication when compared to aceclofenac, aspirin, and bromfenac. However, ibuprofen was inferior to bromfenac (100 mg). The comparison between ibuprofen and diclofenac, metamizole, naproxen sodium, low doses of bromfenac, and ketoprofen showed no differences (Figure 3). The overall evaluation supports the previous outcomes of SPID, TOTPAR, and rescue analgesics taken meta-analysis (Figure 4). 

### 3.4. Adverse Effects

**Figure 5:**
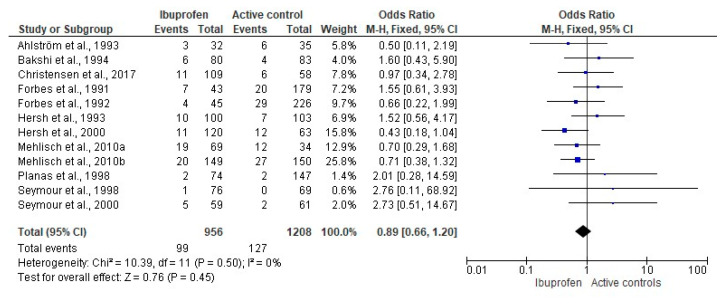
Pooled comparison of adverse effects after third molar surgery.

The global assessment of adverse effects included 12 scientific reports (*n* = 2164). The result of this evaluation indicated that the adverse effects were similar between ibuprofen and other traditional NSAIDs [23,24,25,26,27,28,29,31,32,35,36,37] (Figure 5). 

## 4. Discussion

The primary outcomes of this study showed that ibuprofen 400 mg is more effective than acetaminophen 1000 mg to relieve pain following third molar extraction. Likewise, ibuprofen 200 mg was better than acetaminophen 1000 mg for the management of post-operative pain following third molar removal. These findings are supported by a meta-analysis informing that ibuprofen 200 to 512 mg is a better analgesic treatment than acetaminophen 600 to 1000 mg in mandibular wisdom teeth removal [15]. That review included in the statistical analysis the data of the clinical trial by Mehlisch et al., 1995, which included a comparison between ibuprofen-lysine combination and acetaminophen [38]. That is, the individual effect of ibuprofen was not evaluated, but rather that of the combination of drugs versus acetaminophen. For this reason, this article by Mehlisch et al., 1995, was not included in our review. 

In this same regard, ibuprofen has been shown to be a better analgesic than acetaminophen in different surgical fields. Thybo et al., 2019, demonstrated that an ibuprofen 400 mg—acetaminophen 1000 mg combination had a superior analgesic efficacy (reduction of the morphine post-operative intake) than acetaminophen 1000 mg alone for pain control in total hip arthroplasty. However, the comparison of this drug combination was similarly effective to ibuprofen 400 mg alone for the management of pain following total hip arthroplasty [39]. Kamondetdecha and Tannirandorn (2008) reported that ibuprofen 400 mg offered a better analgesic effect than acetaminophen 1000 mg after childbirth [40]. Ekinci et al., 2020, carried out a randomized, double-blind clinical trial to assess post-operative pain relief using ibuprofen 800 mg, acetaminophen 1000 mg, and placebo in laparoscopic cholecystectomy surgery. The authors observed that ibuprofen was better than acetaminophen [41]. Ciftci et al., 2019 found that an ibuprofen 800 mg group showed low pain scores and minor rescue analgesic intake when compared to the acetaminophen 1000 mg group after laparoscopic sleeve gastrectomy [42]. Erdogan-Kayhan et al., 2018, reported that ibuprofen 400 mg reduced the pain intensity scores in comparison to acetaminophen 1000 mg in bariatric surgery [43]. 

The pooled primary and secondary endpoints evaluation of the analgesic efficacy of ibuprofen 400 mg and diclofenac 50 mg showed no difference. However, the data trend was in favor of diclofenac. It would be important to increase the number of randomized double-blinding clinical trials using a dose of ibuprofen and diclofenac to obtain a definitive conclusion on the analgesic efficacy of these drugs in this kind of surgical procedure. In this same sense, a multiple-dose study showed no difference between these drugs after third molar surgery [44]. Moreover, Gazal and Al-Samadani (2017) demonstrated that ibuprofen had an inferior analgesic activity than diclofenac for dental removal and deep cavity preparation [45].

According to the secondary outcomes, ibuprofen 400 mg reduces the number of patients requiring rescue analgesic medication in the post-operative period when compared to aspirin 650 mg, aceclofenac 150 mg, and bromfenac 5 and 10 mg. Nevertheless, Moore et al., 2015, demonstrated through an indirect comparison that ibuprofen 400 mg is better than aspirin 1000 mg for dental pain management [46]. 

In this review and meta-analysis, we observed a similar risk of adverse effects between ibuprofen and other NSAIDs following wisdom teeth surgery. This is consistent with the findings previously reported by various authors. Southey et al., 2009, and Tan et al., 2020, showed that ibuprofen and acetaminophen have similar safety profiles in pediatric patients [47,48]. van Walsem et al., 2015, found that the risk of fatal and nonfatal severe events was similar between diclofenac, acetaminophen, naproxen, ibuprofen, celecoxib, and etoricoxib. The authors observed a decreased risk of superior gastrointestinal adverse effects using diclofenac when compared with ibuprofen and naproxen in patients with osteoarthritis or rheumatoid arthritis [49]. It is worth noting that the clinical trials included in this review did not find fatal or severe adverse events.

Overall, this study was conducted using only high-quality randomized clinical trials employing rigorous methods [50,51,52]. The most important weakness was that ibuprofen was compared with many active treatments and different doses of these drugs, which made a combined data analysis impossible on many occasions [51,52]. Another disadvantage is that only single-dose studies were included. A multiple-dose scheme is more closely related to the real use of NSAIDs [53].

In conclusion, the synthesis of information and statistical analysis carried out here is intended to be a guide in the choice of one-dose analgesics in third molar surgery. Used under this scheme, ibuprofen 400 mg appears to have good analgesic efficacy and a safety profile similar to other traditional NSAIDs after third molar surgery.

## Data Availability

Data is contained within the article.

## Supplementary Materials

The following are available online at https://www.mdpi.com/article/10.3390/ph14040360/s1, Table S1: Features summary of the high-quality studies. Table S2: Meta-analytical evaluation of the sum of pain intensity differences at 2 and 6 post-surgical hours. Table S3: Pooled assessment of the total pain relief at 2, 4, 6, and 8 h after surgery.
