# Supplementary material for: A Meta-Analysis of the Analgesic Efficacy of Single-Doses of Ibuprofen Compared to Traditional Non-Opioid Analgesics Following Third Molar Surgery
Source: Pharmaceuticals (Basel). 2021 Apr 14;14(4):360. doi: 10.3390/ph14040360 (PMC8070746; doi:10.3390/ph14040360)
Supplement: Supplementary file 1 [file pharmaceuticals-14-00360-s001.pdf]

Review

A Meta-Analysis of the Analgesic Efficacy of Single-Doses of Ibuprofen Compared to Traditional Non-Opioid Analgesics Following Third Molar Surgery

Lorenzo Franco-de la Torre [1], Norma Patricia Figueroa-Fernández [2], Diana Laura Franco-González [1], Ángel Josabad Alonso-Castro [3], Federico Rivera-Luna [2] and Mario Alberto Isiordia-Espinoza [1,*]

1 Instituto de Investigación en Ciencias Médicas, Departamento de Clínicas, División de Ciencias Biomédicas, Centro Universitario de los Altos, Universidad de Guadalajara, Tepatitlán de Morelos, 47620, Mexico; lorfran8888@hotmail.com (L.F.-d.T.); diana.franco5288@alumnos.udg.mx (D.L.F.-G.)
2 Departamento de Cirugía Oral y Maxilofacial, Facultad de Odontología, Universidad Autónoma de Baja California, Campus Mexicali, 21040, Mexico; nfigueroa@uabc.edu.mx (N.P.F.-F.); rivera_l03@hotmail.com (F.R.-L.)
3 Departamento de Farmacia, División de Ciencias Naturales y Exactas, Universidad de Guanajuato, 36250, Mexico; angeljosabad@hotmail.com
* Correspondence: mario.isiordia162@yahoo.com; Tel.: 52-378-119-5786.

Table S1. Features summary of the high-quality studies.

First author, study design and, and analgesic strategy	Treatments (n)	Patients, evaluation period, and number of molars extracted	Evaluation of the clinical efficacy	Adverse effects	Conclusion
Ahlström et al., 1993<br>Randomized, double-blind, parallel, clinical trial.<br>Single-dose study.<br>Post-operative analgesia.	Group A: Ibuprofen 400 mg (n=32).<br>Group B: Diclofenac 50 mg (n=35).<br>Group C: Placebo (n=30).<br>All treatments were administered using the oral route.	Healthy patients.<br>Assessment period was 6 h.<br>A mandibular third molar removed.	Pain was evaluated by VAS at 20 and 40 minutes as well as 1 to 6 post-surgical h.	Diclofenac presented a high incidence of adverse effects when compared to ibuprofen and placebo	Ibuprofen and diclofenac produced similar pain relief-analgesia.

Bakshi et al., 1994. Randomized, double-blind, parallel, clinical trial. Single-dose study. Post-operative analgesia.	Group A: Ibuprofen 400 mg (n=80). Group B: Diclofenac 50 mg (n=83). Group C: Placebo (n=82). All treatments were administered using the oral route.	Healthy patients. Assessment period was 6 h. One mandibular third molar extracted.	Pain at 20 and 40 minutes, as well as 1 to 6 post-surgical h with VAS, and VRS. Other variables were TOTPAR-6, and global evaluation of treatments.	Similar adverse effect were observed for all treatments	Diclofenac produced rapid onset of analgesic effect in comparison with ibuprofen and placebo.
Christensen et al., 2017. Randomized, double-blind, parallel, clinical trial. Single- and multi-dose study. Post-operative analgesia.	Group A: Ibuprofen 600 mg (n=109). Group B: Naproxen sodium 220 mg (n=58). Group C: Placebo (n=29). (Clinical study 1).	Good health patients. The evaluation period was 24 h. A third molar surgery.	Pain relief, PID, SPID, time to confirmed first perceptible relief, time to meaningful pain relief, time to treatment failure, and percentage of treatment failures.	Eleven patients receiving ibuprofen and six patients taking naproxen sodium had adverse effects.	Ibuprofen presented faster onset and superior analgesia than naproxen sodium.
Forbes et al., 1991. Randomized, double-blind, parallel, clinical trial. Single-dose study. Post-operative analgesia.	Group A: Ibuprofen 400 mg (n=37). Group B. Aspirin 650 mg (n=41). Group C: Bromfenac 5 mg (n=39). Group D: Bromfenac 10 mg (n=43). Group E: Bromfenac 25 mg (n=42). Group F: Placebo (n=39).	Patients without clinical significand condition. One to four third molar extractions. Patients were evaluated during 8 h after surgery.	PID-4, PID-8, SPID-4, SPID-8, peak PID score 4 and 8 h, pain relief, TOTPAR-4, TOTPAR-8 peak pain relief, total hours of 50% relief, and overall evaluation.	Similar number of adverse effects were observed in all groups.	Bromfenac 25 mg and ibuprofen 400 mg were significantly superior to the other active treatments.

Forbes et al., 1992. Randomized, double-blind, parallel, clinical trial. Single-dose study. Post-operative analgesia.	Group A: Ibuprofen 400 mg (n=38). Group B. Aspirin 650 mg (n=38). Group C: Bromfenac 10 mg (n=43). Group D: Bromfenac 25 mg (n=41). Group E: Bromfenac 50 mg (n=42). Group F: Bromfenac 100 mg (n=40) Placebo (n=38).	Patients without clinical significand condition. One to four third molar extractions. Patients were evaluated during 8 h after surgery.	PID, SPID, peak PID score, pain relief, TOTPAR-8, peak pain relief, total hours of 50% relief, and overall evaluation.	Similar frequency of adverse effects were observed in all groups.	Bromfenac 100 mg was more effective than ibuprofen.
Hersh, et al., 1993. Randomized, double-blind, parallel, clinical trial. Single- and multi-dose study. Post-operative analgesia.	Group A: Ibuprofen 200 mg (n=51). Group B: Ibuprofen 400 mg (n=49). Group C: Meclofenamate 50 (n=51). Group D: Meclofenamate 100 (n=52). Group E: Placebo (n=51).	Patients with good health. One or more third molar surgeries. Patients were evaluated 8 h following surgery.	Pain intensity evaluation, PID, SPID, peak PID score, pain relief, TOTPAR-8, peak pain relief, overall evaluation, and time to remedication.	Similar number of adverse effects were recorded.	The high dose of ibuprofen and meclofenamate were most effective than the lower doses of these agents after third molar surgery.
Hersh, et al., 2000. Randomized, double-blind, parallel, clinical trial. Single- and multi-dose study. Post-operative analgesia.	Group A: Ibuprofen 200 mg. Group B: Ibuprofen 400 mg. Group C: Acetaminophen 1000 mg. Group D: Placebo.	Patients with good health. One or more third molar surgeries. Patients were evaluated 6 h following surgery.	TOTPAR-2, TOTPAR-6, SPID-2, SPID-6, onset of analgesia, and peak analgesia effect, remedication, and overall evaluation.	All treatments were well tolerated, with no serious side effects reported.	Ibuprofen (200 and 400 mg) was superior to acetaminophen.

Joshi et al., 2004.<br>Randomized, double-blind, parallel, clinical trial.<br>Single-dose study.<br>Pre-operative analgesia.	Group A: Ibuprofen 600 mg (n=31).<br>Group B: Diclofenac 100 mg (n=29).<br>Group C: Paracetamol 1000 mg and Codeine 60 mg (n=30).<br>Group D: Placebo (n=29).	Patients ASA I and II.<br>A third molar extraction.<br>The evaluation period was 24 h.	Pain intensity was assessed using the VAS and VRS.<br>Time for the first analgesic and number of analgesic in the post-operative period were evaluated.	There was no significant difference among the groups.	There was not difference between active treatments.
Mehlisch et al., 2010a.<br>Randomized, double-blind, parallel, clinical trial.<br>Single-dose study.<br>Post-operative analgesia.	Group A: Ibuprofen 400 mg (n=69).<br>Group B: Acetaminophen 1000 mg (n=34).<br>Group C: Ibuprofen 400 mg/acetaminophen 1000 mg (n=67).<br>Group D: Ibuprofen 200 mg / acetaminophen 500 mg (n=33).<br>Group E: Placebo (n=31).	Healthy patients.<br>Two or more third molar surgeries.<br>Assessment period was 8 h.	SPIDR-4, SPRID-6, SPRID-8, TOTPAR-4, TOTPAR-6, TOTPAR-6, re-medication, and global evaluation.	Similar frequency of adverse effects between all treatments.	Ibuprofen/acetaminophen combination produced better analgesia than ibuprofen or acetaminophen alone.
Mehlisch et al., 2010b.<br>Randomized, double-blind, parallel, clinical trial.<br>Single-dose study.<br>Post-operative analgesia.	Group A: Ibuprofen 400 mg (n=74).<br>Group B: Ibuprofen 200 mg (n=75).<br>Group C: Acetaminophen 1000 mg (n=74).<br>Group D: Acetaminophen 500 mg (n=76).<br>Group E: Ibuprofen 400 and acetaminophen 1000 mg (n=149).	Healthy patients.<br>At least 3 lower third molar surgeries.<br>Assessment period was 8 h.	SPID-4, SPID-6, SPID-8, TOTPAR-4, TOTPAR-6, TOTPAR-6, re-medication, and overall assessment of study medication.	There was no significant difference among the groups.	Ibuprofen/acetaminophen mixture was more effective than either drug alone.

	Group F: Ibuprofen 200 mg and acetaminophen 500 mg (n=143).<br>Group G: Ibuprofen 100 mg and acetaminophen 250 mg (n=71).<br>Group H: Placebo (n=73).				
Morrison et al., 2000.<br>Randomized, double-blind, parallel, clinical trial.<br>Single-dose study.<br>Post-operative analgesia.	Group A: Ibuprofen 400 mg (n=127).<br>Group B: Naproxen sodium 550 mg (n=140).<br>Group C: Placebo (n=275).	Healthy patients and patients with controlled systemic disease.<br>At least one mandibular third molar surgery.<br>The evaluation was done through 24 hours.	TOTPAR, onset of analgesia, and peak analgesia effect, and overall evaluation.	Adverse effects of ibuprofen, naproxen sodium, and placebo were no shown.	Rofecoxib provides analgesic efficacy similar a naproxen sodium.
Olson et al., 2001.<br>Randomized, double-blind, parallel, clinical trial.<br>Single-dose study.<br>Post-operative analgesia.	Group A: Ibuprofen 400 mg (n=67).<br>Group B: Ketoprofen 25 mg (n=67).<br>Group C: Acetaminophen 1000 mg (n=66).<br>Group D: Placebo (n=39).	Healthy patients.<br>One or more third molar surgeries.<br>Duration of trial was 6 hours	PRID, TOTPAR-2, TOTPAR-6, SPID-2, SPID-6, SPRID-2, SPRID-6, and the patient's overall evaluation of the study medication.	According to the treatments, the adverse effects were next:<br>5.1% who received placebo.<br>7.5% in ketoprofen. 10.4% in ibuprofen	Ibuprofen was superior to all treatments.

				15.2% in acetaminophen.	
Planas et al., 1998.<br>Randomized, double-blind, parallel, clinical trial.<br>Single-dose study.<br>Post-operative analgesia.	Group A: Ibuprofen 600 mg (n=74).<br>Group B: Metamizol 1000 mg (n=75).<br>Group C: Metamizol 2000 mg (n=72).<br>Group D: Placebo (n=32).	Patients with sufficient mental status to complete the assessment<br>A third molar surgery.<br>Trial duration was 1 hour.	Pain was evaluated using VAS and the verbal scale.<br>PID and SPID were calculated.	There was no difference between treatment groups.	Metamizol 2000 mg was superior to ibuprofen and placebo for control of pain after surgery.
Seymour et al., 1998.<br>Randomized, double-blind, parallel, clinical trial.<br>Single-dose study.<br>Post-operative analgesia.	Group A: Ibuprofeno 400 mg (n=76).<br>Group B: Aceclofenac 150 mg (n=71).<br>Group C: Placebo (n=70).	Healthy patients or patients with controlled systemic disease.<br>One or more third molar extractions.<br>Patients were evaluated 6 hours following surgery.	AUC pain, AUC pain relief, scape analgesic intake, and overall evaluation.	There was no difference between treatment groups.	Ibuprofen was better analgesic than aceclofenac for management of pain after oral surgery.
Seymour et al., 2000.<br>Randomized, double-blind, parallel, clinical trial.<br>Single-dose study.<br>Post-operative analgesia.	Group A: Ibuprofen 200 mg (n=59).<br>Group B: Ketoprofen 12.5 mg (n=61).<br>Group C: Placebo (n=60).	Healthy patients (ASA I).<br>One or more third molar removal.<br>Patients were evaluated 6 hours following surgery.	VAS, VRS, PID-1, SPID-1, peak PID score, pain relief, AUC-4, AUC-6, TOTPAR-4, TOTPAR-6, and peak pain relief.	Similar number of adverse effects were recorded.	Ketoprofen was better than ibuprofen for control of pain.

Table S2. Meta-analytical evaluation of the sum of pain intensity differences at 2 and 6 post-surgical hours.

Subgroups and Authors	Ibuprofen Mean/SD/n	Active Controls Mean/SD/n	Sample Size (n)	OR (95% ICs)	I²%/*p*-Value */Favorable to
**Sum Pain Intensity Differences at 2 h After Surgery**					
Ibuprofen 400 mg versus acetaminophen 1000 mg					
- Hersh et al., 2000	2.65/0.15/59	2.22/0.18/63	122	0.43 (0.37 to 0.49)	
- Olson et al., 2001	3.67/1.2/67	2.8/1.5/66	133	0.87 (0.41 to 1.33)	
Sample size (n)	126	129	255	0.44 (0.38 to 0.50)	71%/0.00001/Ibuprofen
Ibuprofen 400 mg versus ketoprofen 25 mg					
- Olson et al., 2001	3.67/1.2/67	3.43/1.1/67	134	0.24 (−0.15 to 0.63)	NA/0.23/No difference
Ibuprofen 200 mg versus acetaminophen 1000 mg					
- Hersh et al., 2000	2.52/0.16/61	2.22/0.18/63	124	0.30 (0.24 to 0.36)	NA/0.00001/Ibuprofen
**Sum Pain Intensity Differences at 6 h Following Surgery**					
Ibuprofen 400 mg versus acetaminophen 1000 mg					
- Hersh et al., 2000	8.07/0.5/59	5.05/0.58/63	122	3.02 (2.83 to 3.21)	
- Olson et al., 2001	11.77/4.2/67	8.36/4.7/66	133	3.41 (1.89 to 4.93)	
Subtotal sample size (n)	126	129	255	3.03 (2.84 to 3.22)	0%/0.00001/Ibuprofen
Ibuprofen 400 mg versus ketoprofen 25 mg					
- Olson et al., 2001	11.77/4.2/67	9.64/4.4/67	134	2.13 (0.67 to 3.59)	NA/0.004/Ibuprofen
Ibuprofen 200 mg versus acetaminophen 1000 mg					

-	Hersh et al., 2000	6.93/0.59/61	5.05/0.58/63	124	1.88 (1.67 to 2.09)	NA/0.00001/Ibuprofen

SD = Standard deviation; n = sample size; NA = Not applicable; * = Test for overall effect Z.

Table S3. Pooled assessment of the total pain relief at 2, 4, 6, and 8 h after surgery.

Subgroups and Authors		Ibuprofen Mean/SD/n	Active Controls Mean/SD/n	Sample Size (n)	OR (95% ICs)	I²%/*p*-Value */Favorable to
**Total Pain Relief at 2 h After Surgery**						
Ibuprofen 400 mg versus acetaminophen 1000 mg						
-	Hersh et al., 2000	5.6/0.18/59	5.02/0.29/63	122	0.58 (0.49 to 0.67)	
-	Olson et al., 2001	5.51/1.6/67	4.49/2/66	133	1.02 (0.40 to 1.64)	
Sample size (n)		126	129	255	0.59 (0.50 to 0.67)	48%/0.00001/Ibuprofen
Ibuprofen 400 mg versus ketoprofen 25 mg						
-	Olson et al., 2001	5.51/1.6/67	5.27/1.4/67	134	0.24 (−0.27 to 0.75)	NA/0.36/No difference
Ibuprofen 200 mg versus acetaminophen 1000 mg						
-	Hersh et al., 2000	5.39/0.21/61	5.02/0.29/63	124	0.37 (0.28 to 0.46)	NA/0.00001/Ibuprofen
**Total Pain Relief at 4 Post-Operative Hours**						
Ibuprofen 200 mg versus ketoprofen 12.5 mg						

-	Seymour et al., 2000	5.34/5/59	8.47/5.18/61	120	−3.13 (−4.95 to −1.31)	NA/0.0008/Ketoprofen
**Total Pain Relief at 6 h Following Surgery**						
Ibuprofen 400 mg versus acetaminophen 1000 mg						
-	Hersh et al., 2000	16.56/0.75/59	11.9/1.01/63	122	4.66 (4.35 to 4.97)	
-	Olson et al., 2001	17.42/5.7/67	13.3/7/66	133	4.12 (1.95 to 6.29)	
Subtotal sample size (n)		126	129	255	4.65 (4.34 to 4.96)	0%/0.00001/Ibuprofen
Ibuprofen 400 mg versus ketoprofen 25 mg						
-	Olson et al., 2001	17.42/5.7/67	15/6.2/67	134	2.42 (0.40 to 4.44)	NA/0.02/Ibuprofen
Ibuprofen 200 mg versus acetaminophen 1000 mg						
-	Hersh et al., 2000	14.72/0.83/61	11.9/1.01/63	124	2.82 (2.50 to 3.14)	NA/0.00001/Ibuprofen
Ibuprofen 200 mg versus ketoprofen 12.5 mg						
-	Seymour et al., 2000	6.43/6.7/59	9.78/6.9/61	120	−3.35 (-5.78 to −0.92)	NA/0.007/Ibuprofen
**Total Pain Relief at 8 Post-Surgical Hours**						
Ibuprofen 400 mg versus meclofenamate 100 mg						

-	Hersh et al., 1993	12.02/1.56/49	10.48/1.26/52	101	1.54 (0.98 to 2.10)	NA/0.00001/Ibuprofen
Ibuprofen 400 mg versus meclofenamate 50 mg						
-	Hersh et al., 1993	12.02/1.56/49	6.8/1.07/51	100	5.22 (4.69 to 5.75)	NA/0.00001/Ibuprofen
Ibuprofen 200 mg versus meclofenamate 100 mg						
-	Hersh et al., 1993	9.2/1.6/51	10.48/1.26/52	103	−1.28 (−1.84 to −0.72)	NA/0.00001/No difference
Ibuprofen 200 mg versus meclofenamate 50 mg						
-	Hersh et al., 1993	9.2/1.6/51	6.8/1.07/51	102	2.4 (1.87 to 2.93)	NA/0.00001/Ibuprofen

SD = Standard deviation; n = sample size; NA = Not applicable; * = Test for overall effect Z.
